# Acquired Idiopathic ADAMTS13 Activity Deficient Thrombotic Thrombocytopenic Purpura in a Population from Japan

**DOI:** 10.1371/journal.pone.0033029

**Published:** 2012-03-12

**Authors:** Masanori Matsumoto, Charles L. Bennett, Ayami Isonishi, Zaina Qureshi, Yuji Hori, Masaki Hayakawa, Yoko Yoshida, Hideo Yagi, Yoshihiro Fujimura

**Affiliations:** 1 Department of Blood Transfusion Medicine, Nara Medical University, Kashihara, Japan; 2 South Carolina Center of Economic Excellence for Medication Safety and Efficacy and the Southern Network on Adverse Reactions (SONAR), South Carolina College of Pharmacy, University of South Carolina, Columbia, South Carolina, United States of America; Leiden University Medical Center, The Netherlands

## Abstract

Thrombotic thrombocytopenic purpura (TTP) is a type of thrombotic microangiopathy (TMA). Studies report that the majority of TTP patients present with a deficiency of ADAMTS13 activity. In a database of TMA patients in Japan identified between 1998 and 2008, 186 patients with first onset of acquired idiopathic (ai) ADAMTS13-deficient TTP (ADAMTS13 activity <5%) were diagnosed. The median age of onset of TTP in this group of patients was 54 years, 54.8% were female, 75.8% had renal involvement, 79.0% had neurologic symptoms, and 97.8% had detectable inhibitors to ADAMTS13 activity. Younger patients were less likely to present with renal or neurologic dysfunction (p<0.01), while older patients were more likely to die during the TTP hospitalization (p<0.05). Findings from this cohort in Japan differ from those reported previously from the United States, Europe, and Korea with respect to age at onset (two decades younger in the other cohort) and gender composition (60% to 100% female in the other cohort). We conclude that in one of the largest cohorts of ai-TTP with severe deficiency of ADAMTS13 activity reported to date, demographic characteristics differ in Japanese patients relative to those reported from a large Caucasian registry from Western societies. Additional studies exploring these findings are needed.

## Introduction

Thrombotic thrombocytopenic purpura (TTP) is a life-threatening generalized disorder and originally defined by classic “pentad”; thrombocytopenia, microangiopthic hemolytic anemia (MAHA), renal impairment, neurological symptoms, and fever [1]. In 1998, two studies identified deficiency of plasma ADAMTS13 (a disintegrin-like and metalloprotease with thrombospndin type 1 motifs 13) activity (ADAMTS13:AC) among persons with TTP [2], [3]. ADAMTS13 cleaves the peptide bond between Thy1605 and Met1606 in the A2 domain of von Willebrand factor (VWF) subunit. VWF is synthesized in vascular endothelial cells and megakaryocytes. Vascular endothelial cell-derived VWF is released into the plasma as unusually large VWF multimers (UL-VWFMs). UL-VWFMs are degraded into smaller size VWF multimers by ADAMTS13. Severe deficiency of ADAMTS13:AC, either congenital or acquired, results in accumulation of UL-VWFMs and formation of platelet thrombi in the microvasculatures. In congenital TTP (Upshaw-Schulman syndrome), ADAMTS13 deficiency is caused by mutations in the ADAMTS13 gene [4]. In contrast, acquired TTP is frequently caused by inhibitory autoantibodies against ADAMTS13 [2], [3]. Most acquired TTP patients have IgG antibodies. In rare cases, IgA and/or IgM antibodies are associated with IgG antibodies [5], [6]. Patients with severe ADAMTS13:AC deficiency present with a lower platelet count and a significantly increased risk of TTP relapse [7]–[10]. Only a few small cohort studies of acquired idiopathic TTP patients characterized by severe ADAMTS13:AC deficiency have been reported previously. These studies characterize TTP with a predilection for the young and female, high rates of renal and central nervous system (CNS) involvement, and a 15% to 20% mortality. The largest cohort of acquired idiopathic (ai)-severely ADAMTS13-deficient TTP patients previously reported is from the Oklahoma TTP Registry (n = 60) [10]. In this study we systematically analyzed the clinical and laboratory features of a large cohort of Japanese patients with acquired idiopathic TTP and who also have severe ADAMTS13:AC deficiency.

## Results

**Figure 1:**
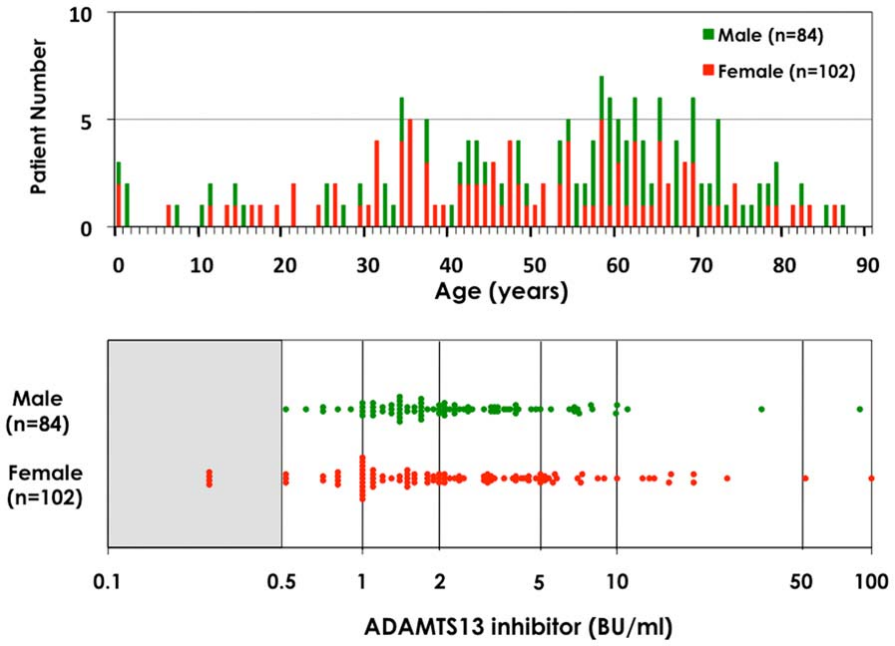
Age distribution and ADAMTS13 inhibitor levels in acquired idiopathic (ai−) TTP with severe deficiency of ADAMTS13 activity. Upper panel shows the age distribution of 186 patients with severe deficiency of ADAMTS13 activity under 5%. We found wide range of the age at TTP bouts from 8 months old to 87 years old. The highest incident peak was found around 60 years old. Lower panel shows the distribution of ADAMTS13 inhibitors in 186 ai-TTP patients with severe deficiency of ADAMTS13 activity. We found ADAMTS13 inhibitors (≥0.5 BU/ml) in 182 patients (97.8%). High titer inhibitors ≥2.0 BU/ml was seen in 101 patients (54.3%).

The number of ai-TTP patients fit the above inclusion criteria and retained for the study was 186. Of these, 31 (16.7%) were diagnosed between 1998 and 2001, 84 (45.2%) between 2002 and 2005, and 71 (38.2%) since 2006. This included individuals who did not experience any exposure to drugs that cause TTP or TMA, organ transplantation, stem cell transplantation, immunologic disease and also did not have a prior history of TTP. The age distribution of disease onset ranged from 8 months to 87 years old, with peak incidence occurring at age 60 (Figure 1, upper panel). Patients under 20 years accounted for 9.1% (17/186) of this subgroup, while patients over age 80 years accounted for 3.8% (7/186). Females accounted for 54.8%. Laboratory studies revealed that 100% of these patients were thrombocytopenic, 75.8% had renal involvement, and 79.0% had neurologic involvement. Overall, 16.1% died from TTP. ADAMTS13 inhibitors (≥0.5 BU/ml) were identified in 182 patients (97.8%). As shown in Figure 1 lower panel, 8.1% of these patients had inhibitor titers of 0.5∼<1.0 BU/ml, 35.5% had titers of 1.0∼<2.0, 33.3% had inhibitor titers of 2.0∼<5.0, 12.9% had inhibitor titers of 5.0∼<10, and 8.1% had inhibitor titers of ≥10 BU/ml. We found four ai-TTP patients without ADAMTS13 inhibitor (<0.5 BU/ml), whose ADAMTS13:AC, however, was normalized after remission. Therefore, these patients were included in this study.

The ai-TTP patients were evaluated according to the age at diagnosis (Table 1); Group 1 (<20 years old: n = 17), Group 2 (20∼<40 years old: n = 36), Group 3 (40∼<60 years old: n = 63), and Group 4 (60 years old∼: n = 70). Rates of renal and neurologic dysfunction at the time of TTP presentation were lowest in the youngest age-subgroup (52.9% versus 72.2% to 81.0% for renal involvement, and 47.0% versus 69.4% to 88.6% for neurologic involvement; p<0.01) while in-patient mortality was highest among the oldest sub-group (28.6% versus 5.9% to 11.1%, p<0.01). Overall, females accounted for 54.8% of the patients (with rates of female gender ranging from 45.7% to 69.4% in each of the four age-groups).

**Table 1:** Clinical features in ai-TTP patients with severe deficiency of ADAMTS13:AC.

	All patients	Groups according to age				
		1	2	3	4	Overall p
Age (years)	54 (37, 65)	<20	20∼<40	40∼<60	60∼	
	Median (25, 75 percentile)					
Patient Number	186	17	36	63	70	
Female (%)	54.8	52.9	69.4	57.1	45.7	NS
“Pentad”						
(1) Platelet count (×10^9^/L), Median (25, 75 percentile)	10 (7, 16)	9 (7, 12)	10 (7, 20)	10 (6, 18)	10 (8, 15)	NS
(2) Hemoglobin (g/dL), Median (25, 75 pecentile)	7.3 (6.1, 8.7)	7.4 (5.4, 8.7)	6.7 (5.9, 7.8)	7.1 (6.0, 8.8)	7.8 (6.6, 8.8)	NS
(3) Renal involvement (%)	75.8	52.9	72.2	81.0	78.5	NS
Serum creatinine (mg/dL), Median (25, 75 percntile)	0.9 (0.7, 1.3)	0.58 (0.31, 0.80)	0.86 (0.70, 1.16)	0.95 (0.80, 1.50)	1.00 (0.80, 1.40)	<0.01^a^
Blood urea nitrogen (mg/dL), Median (25, 75 percntile)	24 (17, 37)	15 (12, 23)	19 (14, 26)	27 (17, 41)	27 (21, 43)	<0.01^b^
(4) CNS involvement (%)	79.0	47.0	69.4	82.5	88.6	<0.01^c^
(5) Fever (≥37.0°C) (%)	71.5	76.5	63.9	69.8	75.7	NS
Mortality in the current episode of TTP bouts (%)	16.1	5.9	5.6	11.1	28.6	<0.05^d^

NS: not significant difference (≥0.05).

Overall p values were caluculated using the Kruskal-Wallis H tests or chi-square tests with Yates' correction for 2×4 tables.

Significant differnces between 4 groups (overall p<0.05) were further analyzed by Mann-Whitney U-test or chi-squre test.

a p<0.01 between Group 1 and Groups 2, 3, 4.

b p<0.01 between Group 1 and Groups 3, 4, and between Group 2 and Groups 3, 4.

c p<0.01 between Group 1 and Groups 3, 4.

d p<0.05 between Group 2 and Group 4.

## Discussion

We evaluated 186 patients with initial onset of severely deficient ADAMTS13:AC levels TTP in Japan, representing the largest cohort of ai-TTP patients with ADAMTS13:AC deficiency reported from Japan. These individuals had presented with TMA-findings to medical centers throughout Japan over a ten-year period. In interpreting our findings, several factors should be considered.

These individuals accounted for 71.5% of 260 patients with a first episode of ai-TTP, who were diagnosed in out registry. This rate is similar to that reported previously for smaller cohorts of TTP patients from Europe, the United States, Canada, the United Kingdom, and Korea [7]–[13].

Sociodemographic characteristics of these TTP patients were compared to those reported from cohorts in Oklahoma, Saint Louis, France, and Korea [7]–[10], [13] (Table 2). The median age of TTP patients with severely deficient ADAMTS13:AC levels reported from the other cohorts except for Saint Louis is 15 to 20 years less than that reported for our cohort. Also, females accounted for 53.8% of patients in our cohort versus 60% to 100% in other cohorts. Since additional information on predisposing factors for TTP are not known currently, it is not possible to identify factors accounting for age- and gender-related differences noted between TTP patients in Japan with severe ADAMTS13-deficiency versus those reported from other geographic regions.

**Table 2:** Comparison of our findings with those reported from Europe, Asia, and the United States for acquired idiopathic TTP patients with severely deficient ADAMTS13:AC levels.

	This study	Vesely et al^7^	Zheng et al^8^	Coppo et al^9^	Kremer-Hovinga et al^10^	Jang et al^13^
	(n = 180)	(n = 16)	(n = 16)	(n = 31)	(n = 60)	(n = 20)
Geographic region	Japan	Oklahoma (USA)	Saint Louis (USA)	France	Oklahoma (USA)	Korea
Ethnicity/race	Japanese 100%	White 50%,	White 32%,	White 52%,	African-American 35%	Korean 100%
		African-American 50%	African-American 68%	Afro-Caribbean 48%		
Idiopathic etiology	100%	100%	100%	100%	77%	70%
Prior TMA	0%	0%	38%	13%	0%	ND
ADAMTS13:AC	<5%	<5%	<5%	<5%	<10%	<10%
ADAMTS13:INH	98%	94%	44%	55%	83%	ND
Age (years)	54 (8 m–87)	39 (19–71)	51 (21–79)	36 (19–67)	41 (9–72)	40.5 (mean)
% female	55	75	100	65	82	60
Platelets (10^9^/ul)	10 (1–88)	11(4–27)	17 (6–47)	12 (2–69)	11 (2–101)	24 (mean)
Hb (g/dl)	7.3 (4.3–11.9)	ND	ND	7.3 (4.6–13.7)	ND	7.7 (mean)
Ht (%)	ND	21 (15–30)	25 (13–33)	ND	21 (13–33)	ND
Creatinine (mg/dl)	0.9 (0.7–10.7)	1.2(0.9–5.5)	1.1 (0.7–3.1)	1.1 (0.67–5.2)	1.6 (0.7–6.6)	1.6 (mean)
BUN (mg/dl)	23.4 (2.5–154)	ND	ND	ND	ND	ND
Fever (%)	72	ND	31	36	ND	70
CNS involvement (%)	79	50	56	74	50	25
% Survival	84	81	81	87	78	81

**ND: no data.**

**Median (minimum-maximum).**

Age-related differences in rates of neurologic and renal involvement among TTP patients who had severely deficient ADAMTS13:AC levels have not been reported previously. We found lower rates of renal and neurologic dysfunction amongst the youngest TTP patients, and the highest short-term mortality rates among the oldest TTP patients. While our study evaluated 186 patients with initial onset of severe ADAMTS13:AC activity deficiency, the other cohorts included smaller number of patients with idiopathic TTP and severe ADAMTS13:AC deficiency [7]–[9], [11]–[13]. These age-related differences in clinical findings may account in part for higher short-term mortality rates observed among older patients with TTP in our cohort, as well as in the cohort reported from Canada [14].

Fourth, inhibitory autoantibodies against ADAMTS13 were identified in 97.8% of patients with ADAMTS13:AC deficient TTP. Other cohorts identify inhibitory antibodies in 44% to 94% of TTP patients with severely deficient ADAMTS13:AC levels [7]–[13]. These findings reflect variable sensitivity and specificity of ADAMTS13:AC inhibitor tests. In our study, ADAMTS13 inhibitor levels of 5 or more BU/ml were identified in 21.0% of TTP patients with severely deficient ADAMTS13:AC levels and inhibitor levels of 10 or more BU/ml were noted in 8.1%. These TTP patients with severely deficient ADAMTS13:AC activity levels and high titer inhibitors to ADAMTS13 might represent a subgroup of TTP patients for whom rituximab therapy might be particularly beneficial [12]. In general, the role of IgG antibody levels in ai-TTP is felt to be controversial. Some investigators report an association of higher titers with increased mortality, refractoriness, and more severe presentation [10], [15], while others have not found similar results [7], [16].

Our study has the limitation that follow-up ended at the time of hospital discharge, which prevented us from reporting on relapse rates and long-term survival rates. A second limitation is that TTP patients who were not severely deficient in ADAMTS13:AC levels were not included in this study. As noted by others, this is a heterogenous group of patients- many of whom have diseases other than TTP. Another limitation is that while our laboratory is a distinguished referral center for TMAs in Japan, it is not mandatory that information on all TMA patients is sent to our laboratory, and hence a number of patients with TMAs in Japan are not entered into our database. A final limitation is that cohorts in two of the five comparison studies (from Korea and Oklahoma) included a minority of individuals with TTP who did not have primary idiopathic TTP [10], [13].

In summary, findings from this cohort of TTP patients in Japan with severe ADAMTS13:AC deficiency parallel those reported from TTP cohorts in Europe, the United States, Canada, the United Kingdom, and Korea in several ways, but also provide insights that have not been reported previously [7]–[14]. Novel findings in this cohort include females accounting for only 54.8% of incident cases, a higher median age at TTP onset of 54 years, and higher mortality rates amongst patients who were older than 60 years of age. Given the rarity of TTP in the general population, aggregation of findings from various TTP cohorts reported from Japan, Korea, France, England, Saint Louis, Oklahoma, and Canada might yield important findings that single registries would be unable to identify. A particularly important finding might be development and validation of a multivariate model predictive of mortality of persons with incident TTP characterized by severe ADAMTS13:AC deficiency.

## Methods

Since 1998, our laboratory has been a nationwide referral center within Japan for TMAs, with 919 patients having been registered in this database [17]. During the first years of the study, samples from all TMA patients were evaluated by our referral center. In recent years, commercial laboratories now provide access to ADAMTS13:AC evaluation and some centers therefore do not submit samples to our group. We are not able to ascertain which centers are sending samples to commercial vendors at this time. Of these 919 patients, 186 patients were diagnosed with first onset of ai-TTP characterized by severe deficiency of ADAMTS13:AC (<5%) and no prior history of TTP. Exclusion criteria were exposure to drugs that cause TTP or TMA, organ transplantation, stem cell transplantation, immunologic disease, or ADAMTS13:AC levels 5% and more. All patients gave written informed consent to participate in this study. The study protocol was approved by the Ethics Committee of Nara Medical University Hospital.

### Diagnostic criteria

The classic pentad for TTP was defined as follows (i) microangiopathic hemolytic anemia (hemoglobin ≤12 g/dL), Coombs test negative, undetectable serum haptoglobin (<10 mg/dL), more than 2 fragmented red cells (schistocytes) in a microscopic field with a magnification of 100, and concurrent increased serum lactate dehydrogenase (LDH) above institutional baseline, (ii) thrombocytopenia (platelet count ≤100×10^9^/L), (iii) fever ≥37°C, (iv) CNS involvement: ranging from headache to coma, including neurological dysfunction, convulsion, clouding of consciousness, and (v) renal involvement (including abnormal urinalysis in addition to elevation of serum creatinine level). Patients were excluded if they reported a prior episode of ai-severely ADAMTS13-deficient TTP (n = 18 patients).

### Blood Sampling

Before therapeutic approaches were initiated, whole blood samples (∼5 ml) were phlebotomized from each patient and placed into plastic tubes containing 1/10 volume of 3.8% sodium citrate. The plasma was separated by centrifugation at 3000 g for 15 min at 4°C, kept in aliquots at −80°C until testing, and sent to our laboratory with clinical information.

### Assays of plasma ADAMTS13:AC and ADAMTS13:INH

Until March 2005, ADAMTS13:AC was determined by classic von Willebrand factor multimer (VWFM) assay with a detection limit of 3% of the normal control [18], [19]. Thereafter, a chromogenic ADAMTS13-act-ELISA [20] with a detection limit of 0.5% of the normal control was developed, and replaced the VWFM assay. Thus, most of the plasma samples stored at −80°C were re-evaluated with chromogenic act-ELISA, but 22 samples were unable to evaluate by the new method, because of a short of the stored sample volume. Basically, however, the results obtained by both the assays had a high correlation (r = 0.99) [20]. Thus, the results determined by VWFM alone were also included in this study. Further, to compare the results from other investigators and potentially with different assay methods for ADAMTS13:AC, we here categorized plasma levels of ADAMTS13:AC of severe (<5%), moderate (5%∼<25%), mild (25%∼<50%) deficiency and normal (≥50%) of ADAMTS13:AC. Plasma ADAMTS13:INH titers were analyzed either by classic VWFM assay or chromogenic ADAMTS13-act-ELISA using heat-inactivated plasmas at 56°C for 30 min. Briefly, the tested samples were mixed with an equal volume of the normal plasmas and incubated at 37°C for 2 hours. After incubation, the residual ADAMTS13:AC was measured. One Bethesda unit (BU) is defined as the amount necessary to reduce ADAMTS13:AC to 50% of control levels according to the Bethesda method, which was originally developed for the measurement of factor VIII inhibitor [21]. Titers ≥0.5 BU/ml were classified as inhibitor-positive.

### Statistical analysis

All continuous variables were reported as median values (25, 75 percentile). Comparisons between two patient groups (severe deficiency and detectable ADAMTS13 activity) were tested for statistical significance using the Mann-Whitney U-tests or chi-square tests. Comparisons between 4 patients groups (under 20 years old, 20 to under 40 years old, 40 to under 60 years old, and over 60 years old) were calculated using the Kruskal-Wallis H tests or chi-square tests with Yates' correction for 2×4 tables. Significant differences between 4 groups (overall p<0.05) were further analyzed by Mann-Whitney U-tests or chi-square tests. Correlation between ADAMTS13:AC and :INH was analyzed by Sperman's correlation. A two-tailed P value less than 0.05 was considered to be significant.
